# Assessment process followed by elementary school teachers in Japan for assessing the support needs of children of parents with cancer: a semi-structured interview

**DOI:** 10.1186/s12887-026-06747-0

**Published:** 2026-03-21

**Authors:** Yuko Akagawa, Kaori Osawa, Takumi Takahashi, Sue P Heiney, Sachiko Makabe

**Affiliations:** 1 Department of Nursing, Akita University Graduate School of Health Sciences, Akita, Japan; 2 Cancer Consultation Support Center, Tokyo Kyosai Hospital, Tokyo, Japan; 3 Department of Nursing, Akita University Hospital, Akita, Japan; 4 College of Nursing, University of South Carolina, Columbia, South Carolina USA

**Keywords:** Parental cancer, Child support, Elementary school, Teacher judgment

## Abstract

**Backgrounds:**

Children of parents with cancer are often affected physically, psychologically, and socially. School teachers are close observers of children’s behavior and emotional stability. Their awareness and judgment are crucial for detecting changes early and linking children to appropriate support. However, the role of school teachers in recognizing these signs and the process by which they determine the need for support remain largely unexplored, particularly in Japan.This study aimed to identify the process by which elementary school teachers in Japan assess the support needs of children whose parents have cancer. The findings of this study contribute to the development of school-based support systems for these children.

**Methods:**

A descriptive phenomenological design was adopted. Semi-structured interviews were conducted with public elementary school teachers in Akita City. The interview guide covered three main areas: (1) situations in which teachers perceived a need for support, (2) the reasons and contextual factors underlying such judgments, and (3) how teachers interpreted subtle signs and behavioral changes in children. Data were analyzed using Colaizzi’s seven-step descriptive phenomenological method to clarify the essential structure of teachers’ decision-making experiences.

**Result:**

Interviews with 24 teachers (conducted between December 2024 and June 2025) revealed four thematic categories: (1) Noticing changes in children, (2) Interpreting those changes and experiencing wavering judgments, (3) Regulating emotional conflicts that hinder offering support; and (4) Recognizing and reaffirming their role as educators in providing support. Taken together, these categories depict a progressively negotiated decision-making structure in which teachers move from intuitive noticing to reflective interpretation and role-based action. The findings clarify that support-related judgments are shaped by ethical hesitation, uncertainty, and gradual consolidation of professional responsibility rather than immediate assessment.

**Conclusion:**

The complexity of decision-making in schools regarding support for children of parents with cancer highlights the need for close collaboration with health care professions. Observations and insights gained by teachers through daily interactions can be valuable for the entire health care team, and such collaborations can enhance support quality. Therefore, developing interprofessional systems and educational initiatives that foster mutual understanding of professional roles may enhance timely and comprehensive support for these children.

**Supplementary Information:**

The online version contains supplementary material available at 10.1186/s12887-026-06747-0.

## Background

Cancer is a major health issue in Japan, with approximately one million new cases diagnosed annually as of 2021 [1]. Data from the National Cancer Center Hospital indicate that 24.7% of 6,700 inpatients aged 20–59 years had children younger than 18 years from 2009 to 2013, implying that one in four patients with cancer raises children while receiving treatment [2]. Nationwide estimates suggest that approximately 56,000 patients with cancer annually are parents to approximately 87,000 children aged < 18 years [2]. The mean age of fathers and mothers with cancer is 46.6 and 43.7 years, respectively, whereas their children are aged 11.2 years on average, with more than half of these children being aged 0–12 years [2]. Parental cancer occurs during a life stage in which parents bear important social and familial responsibilities, and their children are still undergoing growth and development. Consequently, parental cancer imposes a major psychological and economic burden on the entire family.

Changes in family atmosphere and daily routines often occur following a diagnosis of parental cancer due to treatment-related disruptions, physical and psychological instability, and shifting roles within the household [3–5]. These changes may affect the relationship between parents and their children, reduce communication within the family, and create a stressful home environment [6, 7]. Such environmental disruptions are burdensome for school-aged children because they may experience difficulty in accurately understanding a parent’s illness or struggle to verbalize their anxiety and confusion. Additionally, they are prone to internalizing emotions [8], leading to reduced concentration in schoolwork, unstable peer relationships, somatic complaints, and emotional instability [9–11]. Facing parental cancer at this developmental stage can profoundly affect developmental tasks and school adjustment, making early recognition and support essential.

In this context, teachers hold a unique position in the school setting, where they can promptly and continuously observe behavioral and emotional changes in children, including changes in their expressions and speech. Nonetheless, little is known about how teachers interpret these changes and determine the need for support. Moreover, teachers are primarily educators but are sometimes expected to act as caregivers. This role ambiguity and the associated conflicts [12] may influence their judgment and actions regarding support. Furthermore, although the basic awareness of teachers and the importance of support for children with parents with cancer have been highlighted in prior research [12–14], evidence remains limited regarding the practical aspects of how teachers determine the necessity of support and the processes through which they proceed toward providing it.

The recognition and decision-making processes underlying teachers’ awareness of children’s needs should be clarified to address the challenges in supporting school-aged children with parents with cancer. Such knowledge may offer valuable insights into the appropriate timing of support initiation and approaches to family support by healthcare professionals, including nurses. Fostering mutual understanding and information sharing between educational settings (where teachers interact with children daily) and healthcare settings (where treatment and care are provided) may lay the foundation for comprehensive support for school-aged children with parents with cancer.

Therefore, this study aimed to clarify the process through which elementary school teachers determine the necessity of support for children with parents with cancer. Understanding this process has valuable implications for healthcare professionals, including nurses, in detecting changes in children in the school setting and determining the appropriate timing and strategies for family support.

## Methods

### Study design

Using data obtained through one-on-one semi-structured interviews with teachers, descriptive phenomenology sought to clarify how elementary school teachers determined the necessity of support for children with parents with cancer and how they proceeded to take action. A descriptive phenomenology [15, 16] was considered appropriate for this study to gain an in-depth understanding of the subjective processes by which individual teachers observed behavioral and emotional changes in children and recognized the need for support based on their backgrounds, values, and practical experiences.

### Participants

This study used purposive sampling and included elementary school teachers working at public schools in Akita City who had experience supporting children with parents with cancer. No restrictions were imposed on age or gender. However, teachers whose study participation was anticipated to result in psychological distress due to the study theme (cancer and support) were excluded.

### Data collection

The first author, who has clinical experience in oncology nursing at a specialized cancer hospital and has been involved in counseling and support programs for parents with cancer for approximately 10 years [17–19], conducted the interviews from December 2024 to June 2025 and conducted a cross-sectional survey to ascertain the number of cases supported by elementary school teachers and to explore their perceptions, knowledge, and concerns regarding support for children with parents with cancer [12]. The survey included an invitation to participate in a follow-up qualitative interview. Teachers who indicated their willingness and contacted the research team were purposively selected for the interview phase. This recruitment strategy ensured that participants had direct and relevant experience related to the research aim. Regarding the sample size, in accordance with descriptive phenomenological methodology, experiential depth and the elucidation of essential meaning structures were prioritized over numerical representativeness. The narratives obtained from the 24 participants provided sufficient informational richness to clarify the essential structure of teachers’ decision-making experiences.

One-on-one interviews were conducted online according to the participants’ preferences, with each interview lasting approximately 40–60 min. All interviews were audio-recorded with participants’ consent.

Prior to the interview, the participants were asked to provide the following demographic data: (i) age, (ii) years of teaching experience, (iii) position (classroom teacher, administrator, school nurse, etc.), (iv) personal or family experience with cancer, (v) experience in cancer education, and (vi) number of cases in which they provided support to children with parents with cancer.

The semi-structured interview guide included the following open-ended questions: (i) “In what situation did you first feel that ‘support might be needed’ for children with parents with cancer, and what aspects of the children’s condition or changes were most striking at that time?” (ii) “What were the decisive reasons or contextual factors for considering the necessity of support? Please describe any emotions or hesitations you felt at that time.” (iii) “How did you, as a teacher, interpret any discomfort or signs you noticed while interacting with the children?” During the interviews, the participants were encouraged to speak freely about their experiences, and probing and follow-up questions were used as needed to achieve an in-depth understanding and improve the data reliability.

The semi-structured interview guide was newly developed for this study and is provided as Supplementary File 1.

Data collection continued until conceptual saturation was achieved. Saturation was defined as the point at which no new thematic insights or structural elements of the decision-making experience emerged in consecutive interviews and when the essential experiential structure could be clearly articulated. This determination was supported through ongoing analytic discussions among the research team.

### Data analysis

Data were analyzed using Colaizzi’s phenomenological method to capture the essence of the participants’ experiences and meanings [20]. This method can clarify the underlying structures and meanings of subjective experiences and is widely used in qualitative research in the fields of education and nursing. The analytic process proceeded as follows: (i) *familiarization with transcripts*: all interview recordings were transcribed verbatim and carefully read to gain an overall understanding; (ii) *extraction of significant statements*: descriptions and expressions relevant to the study aim were extracted; (iii) *formulation of meanings*: the researchers rephrased the extracted statements into plain expressions based on the context to formulate meanings; (iv) *clustering of meanings and development of categories*: the formulated meanings were grouped according to similarity, and initial categories and subcategories were generated; (v) *development of comprehensive descriptions*: the experiences of the participants were described in detail based on subcategories; (vi) *extraction of fundamental structure*: the essential structure related to the teachers’ decision-making process regarding support was articulated; (vii) *member checking*: the preliminary results were shared with co-researchers (a nurse and a social worker), and several participants, and feedback on interpretations and necessary modifications were obtained to enhance the validity of findings.

Throughout the analytic process, discussions with co-researchers were conducted to analyze the appropriateness of the themes and codes, and efforts were made to minimize interpretive bias. To enhance phenomenological rigor, reflexive bracketing was undertaken throughout the analytic process. Given the first author’s clinical background in oncology nursing and prior involvement in support programs for families affected by cancer, she documented her pre-understandings and assumptions before and during analysis. These reflections were continuously examined through discussions with co-researchers to minimize interpretive bias and maintain openness to participants’ lived experiences.

### Ethical considerations

This study was approved by the research ethics committee of the first author’s affiliated institution (approval no. 3112). The participants were provided with written and verbal explanations of the study aim and methods, voluntary participation, privacy protection, management of recorded data, and scope of academic use. Written informed consent was obtained after the participants agreed to participate and demonstrated sufficient understanding.

Participation was entirely voluntary, and the participants were informed that they could withdraw at any time, even after the study commenced. Additionally, the participants were informed in advance that recalling past experiences and emotions during the interviews might cause psychological distress, and they were assured that any interview could be discontinued at any time upon request.

All data were anonymized to prevent individual identification, and pseudonyms and numbers were used in the transcripts. Data were securely stored by the principal investigator.

## Results

Semi-structured interviews were conducted with 24 elementary school teachers aged 45.5 years (median). The characteristics of the participants are summarized in Table 1. All participants were classroom teachers, with female and male teachers accounting for 70.8% (*n* = 17) and 29.2% (*n* = 7), respectively. The median teaching experience was 20 years, and the median number of cases in which the participants supported children with parents with cancer was two.

**Table 1:** Participant Characteristics (*N* = 24)

Category	Details	Number (%)	
Position	Homeroom teacher Assistant teacher	22(91.7) 2(8.3)	
Gender	Female Male	17(70.8) 7(29.2)	
Age (median)			45.5 years (range: 32–62)
Years of teaching experience (median)			20 years (range: 5–38)
Experience providing cancer education	Yes No	2(8.3) 22(91.7)	
Personal or family cancer experience	Yes No	12(50.0) 12(50.0)	
Number of support experiences (median)			2 cases (range: 1–4)

Regarding the process by which elementary school teachers determined the necessity of support for children, four major categories were identified (Table 2). Through daily observations, the teachers detected and interpreted subtle changes in the children, and they wavered in their judgment about whether to take supportive action. This sequence of processes involves multiple interrelated factors. The following subsections present the categories, corresponding subcategories, and illustrative quotes.

**Table 2:** Assessment process used by elementary school teachers to determine the necessity of support for children whose parents had cancer

Category	Subcategory
Noticing changes in children	Emergence of physical symptoms
	Disruptions in behavioral patterns
	Fragmented verbal expressions
	Changes in peer relationships
	Fluctuations in emotional expression
Interpreting changes and having wavering judgment	Observational judgment of transient versus persistent changes
	Between speculation and certainty regarding family circumstances
	Determining the timing of support initiation
Regulating emotional conflicts that hinder the provision of support	Desire to wait for children’s disclosure
	Hesitation to intrude into family matters
	Cautiousness about interventions and awareness of responsibilities
Recognizing and reaffirming the role of teachers as educators in the provision of support	Awareness as a trusted recipient within relationships of trust
	Supportive perspective of monitoring children’s changes in daily life
	Recognizing the limits of teachers and expectations for collaboration

The first category was “Noticing changes in children.” As the starting point for determining the necessity of support, teachers focused on changes in the daily behaviors and attitudes of children. These manifestations varied according to their developmental stage (lower, middle, and upper grades).

### Emergence of physical symptoms

Children in the lower grades sometimes express unarticulated anxiety or stress, as manifested by somatic complaints such as stomachaches and headaches. Teachers intuitively perceived that these symptoms reflected changes in the home environment.

*“When I was a first-grade homeroom teacher*,* one child all of a sudden began saying every morning*,* ‘My stomach hurts.’ But when they went to the school nurse*,* nothing seemed wrong…. I felt that something was off. Later I heard*,* ‘My mom is in the hospital*,*’ and then it all made sense.” (Teacher A)*.

*“The child kept having a slight fever again and again*,* but it did not really feel like a cold. I thought maybe it was more emotional.” (Teacher D)*.

### Disruptions in behavioral patterns

Children in the middle grades often manifest changes as behavioral fluctuations in their daily routines, such as frequent forgetfulness or delays in submitting assignments. The teachers perceived these as indicative of discomfort.

*“This child used to be really reliable*,* but all of a sudden*,* started forgetting things a lot. That’s when I first thought*,* something’s different.” (Teacher H)*.

*“The child was often late in turning in worksheets*,* and when I asked*,* they quietly said*,* ‘Yesterday*,* my mom was at the hospital….’” (Teacher K)*.

### Fragmented verbal expressions

Children in the middle to upper grades sometimes mentioned the situation of their families in diaries or conversations through fragmented verbal cues. The teachers considered these utterances subtle signs of the need for support.

*“The word ‘hospital’ came up in the diary*,* and I thought*,* ‘Huh*,* that’s odd.’ As we talked a few more times*,* I started to see the situation.” (Teacher U)*.

*“The child wrote*,* ‘My mom went to the hospital again today*,*’ and it went on like that for days.” (Teacher N)*.

*“One day*,* the child suddenly told me*,* ‘My mom’s hair fell out.’ But they did not say anything more…. I felt like they wanted to tell me*,* but couldn’t say it all.” (Teacher E)*.

### Changes in peer relationships

Some children in the upper grades exhibited inner distress, as manifested by changes in their peer relationships, such as distancing themselves from friends and becoming more isolated.

*“The child suddenly stopped talking to the friend they were always with…. It seemed like they were holding it all inside*,* without asking anyone for help.” (Teacher S)*.

*“Another student told me*,* ‘That child hasn’t been in the classroom much lately*,*’ and that’s how I finally noticed.” (Teacher M)*.

### Fluctuations in emotional expression

Children in the upper grades showed emotional instability, shifting between extremes (i.e., expressing emotions intensely [crying or sudden anger] and subsequently suppressing them [remaining expressionless]).

*“There was this sixth-grade boy*,* usually calm*,* but he suddenly started getting angry over little things…. That’s when I began responding together with the homeroom teacher.” (Teacher T)*.

*“A sixth-grade girl suddenly burst into tears during class. When I took her to another room*,* she said*,* ‘Why is it always me?’ That’s when I first thought*,* maybe something was going on at home.” (Teacher N)*.

*“There was a child whose face was always stiff*,* saying nothing*,* but I could just feel that something was there.” (Teacher V)*.

The second category was “Interpreting changes and having wavering judgment.” After noticing changes in children, the teachers carefully judged whether these changes were transient or persistent and cautiously considered the necessity of support.

### Observational judgment of transient versus persistent changes

The teachers initially regarded the children’s conditions as “common complaints.” However, these changes persisted over time, or their quality shifted. Consequently, the teachers increasingly leaned toward recognizing their need for support.

*“At first*,* I just thought it was a simple health problem. But even after a week*,* the child’s condition did not return to normal. That’s when I felt*,* this is a little different.” (Teacher E)*.

*“Kids often say*,* ‘My stomach hurts*,*’ but this child would complain at the same time every single day. It became like a routine*,* and I started to think that maybe this is more psychological.” (Teacher D)*.

### Between speculation and certainty regarding family circumstances

From observing the children’s behaviors, the teachers often sensed that “something was happening at home.” However, it was not always whether these were related to parental cancer, and they struggled to interpret ambiguous cues.

*“I heard from another child*,* ‘It seems her mom is in the hospital*,*’ but the child herself never said anything*,* so I wasn’t sure how to take it.” (Teacher I)*.

*“From the child’s words and behavior*,* I kind of felt that something difficult might be going on at home. But without anything definite*,* I hesitated to ask*,* and I was really torn about it.” (Teacher B)*.

*“The parent once said*,* ‘I am not feeling very well…’ and nothing more. But after that*,* the child’s condition started to change*,* and little by little I began to think that maybe this is something more serious.” (Teacher O)*.

### Determining the timing of support initiation

Deciding when and how to begin supportive actions was a major challenge. The teachers continuously wavered between caution and urgency, struggling with issues such as when to speak up and how much to intervene.

*“I am always torn about whether I should talk to the child or keep watching a little longer. I wonder when the right time is. I know I shouldn’t rush it.” (Teacher F)*.

*“Even when I feel*,* ‘This child does not look well*,*’ it is hard to take action without any information from the parents. But at the same time*,* doing nothing did not feel right either…. I was really conflicted between the two.” (Teacher J)*.

*“If the child says nothing*,* asking directly from my side might actually become a burden. So I kept thinking that waiting can also be a form of support.” (Teacher R)*.

The third category was “Regulating emotional conflicts that hinder the provision of support.” Teachers faced psychological barriers and experienced conflicts that stemmed from their sense of professional responsibility, which influenced their decisions regarding when and how to initiate support.

### Desire to wait for children’s disclosure

Several teachers wished that the children would speak when they were ready. They were concerned that rushing to provide support might intrude too deeply into the children’s inner worlds.

*“I really feel I want to wait until the children talk on their own. Forcing them to tell me does not feel right. In those moments*,* I think each child has his or her own kind of ‘readiness to talk.’” (Teacher L)*.

*“Even if it is a child who cries a lot*,* I wait until they show some sign that they want to say something themselves. If I get the timing wrong*,* I feel like it could actually break the trust.” (Teacher E)*.

*“When a child came to me saying*,* ‘Sensei*,* actually…’ I realized*,* ‘Ah*,* this must be the moment they felt that they are ready to talk.’ Until then*,* I deliberately avoided asking from my side.” (Teacher S)*.

### Hesitation to intrude into family matters

The teachers were hesitant and cautious about becoming involved in private areas, including the situations of children’s families and details of their illnesses. They expressed difficulty in drawing the line between providing support as teachers and interfering in family matters.

*“When it comes to things at home*,* I naturally become very cautious. I struggle with how much I should ask. Especially with illness*,* the child and the parent might not want me to touch on it at all.” (Teacher D)*.

*“It is hard to draw the line inside myself about what falls under the school’s role. Home matters are home matters*,* but if it is affecting the child*,* I cannot just ignore it…. I kept wavering in between.” (Teacher K)*.

### Cautiousness about interventions and awareness of responsibilities

The teachers were acutely aware that there was no single “correct” way to support the children, and they constantly considered the possible impact of their words and actions. Their professional responsibilities, even when acting with goodwill, compel them to act cautiously.

*“I cannot help but think*,* ‘What if the way I get involved has a negative effect….’ As teachers*,* even if we mean it as support*,* we still have to take responsibility for the outcomes. Especially when illness is involved*,* it is not something we can act on lightly.” (Teacher T)*.

*“I always worry that even a small word of encouragement might become pressure for the child or that the parents might feel I’m interfering unnecessarily.” (Teacher M)*.

*“The word ‘support’ sounds nice*,* but if you approach it in the wrong way*,* it can actually hurt the child. When I think about that*,* I can’t help but be cautious.” (Teacher H)*.

The fourth category was “Recognizing and reaffirming the role of teachers as educators in the provision of support.” After observing changes in the children and identifying the necessity of support, the teachers reconsidered their professional duties and expertise and explored means of providing support in the school setting. Based on their sense of responsibility as “the ones who noticed,” the teachers eventually viewed support not as something special but as a part of daily practice. Furthermore, they became increasingly aware of their role as a bridge connecting children and their families with specialized professionals.

### Awareness as a trusted recipient within relationships of trust

The teachers expressed that they had a responsibility to listen attentively to children and take their disclosure of personal matters seriously. The teachers acknowledged that such disclosure required courage on the children’s part to speak about family circumstances, and they felt strongly accountable for being entrusted with such information. Teachers’ receptive attitudes was grounded in trust, became the starting point for support.

*“When a child said*,* ‘I will tell this only to you*,* Sensei*,*’ I felt that it must be something really important. I thought the first thing I needed to do was to listen carefully so as not to betray that trust.” (Teacher S)*.

*“When a child said*,* ‘This is just between us*,*’ I thought*,* ‘Wow*,* this child is really trying hard.’ Sharing something like that is not easy at all.” (Teacher K)*.

### Supportive perspective of monitoring children’s changes in daily life

Rather than focusing solely on formal interventions, the teachers emphasized that attending to small daily changes in children’s behaviors and expressions was an entry point for providing support.

*“I value those small feelings of*,* ‘Something seems different from usual.’ You can only notice that kind of thing if you’re watching the child every day.” (Teacher O)*.

*“Saying a few words*,* watching their facial expressions—those small things*,* one after another*,* are what I see as the first step in providing support.” (Teacher B)*.

### Recognizing the limits of teachers and expectations for collaboration

Teachers were aware of their limitations and recognized the necessity of collaborating with school nurses, counselors, and families. They sought to actively fulfill their role as a bridge rather than attempting to handle the situation alone.

*“It is really impossible for a homeroom teacher to carry everything alone. We needed to work together as a team*,* linking with the school counselor and the school nurse.” (Teacher P)*.

*“After listening to a child*,* I do not feel secure acting only on my own judgment. So I try to move forward by sharing information with other teachers.” (Teacher N)*.

## Discussion

This study focused on the process through which Japanese elementary school teachers determine the necessity of support for children whose parents have cancer. As a result, four categories were identified from the teachers’ narratives—namely, (i) “Noticing changes in children,” (ii) “Interpreting changes and having wavering judgment,” (iii) “Regulating emotional conflicts that hinder the provision of support,” and (iv) “Recognizing and reaffirming the role of teachers as educators in the provision of support.” These categories illustrate the practical and ethical challenges of making support-related judgments in school settings and highlight the potential and necessity of collaboration with healthcare professionals.

### Noticing changes in children: educational perspectives on developmental stages and clinical significance

Upon observing changes in children, teachers carefully identified various signs corresponding to developmental stages, including physical complaints, behavioral disruptions, fragmented verbal expressions, changes in peer relationships, and fluctuations in emotional expression. This finding suggests that psychological signs in children, which are frequently difficult for families to detect, manifest within the school environment, underscoring the value of school-based observation.

This finding is not limited to the context of parental cancer; rather, it reflects a broader issue relevant to children experiencing psychosocial burdens under diverse circumstances. For instance, continuous observation in the school setting is considered a key challenge in supporting young carers who assume responsibilities at home. As demonstrated by previous studies conducted in Japan and other countries, the family related burden and psychological stress experienced by children often remain invisible to others, leading to delays in the initiation of support [21, 22]. Parrillo et al. reported that early recognition and intervention by schools were effective in supporting academic and emotional recovery among children with cancer [23]. Watson et al. showed that children with parents with cancer are vulnerable to emotional and behavioral difficulties [24]. These findings suggest that early recognition through teachers’ observations provides essential information for healthcare professionals to identify family psychosocial risks and develop appropriate support plans.

### Interpreting changes and having wavering judgment: the complexity of inferential judgment in educational settings

The teachers carefully deliberated on whether the changes observed in the children were transient or indicative of the need for sustained support, engaging in repeated observations and reflections. Given the limited availability of information linking such changes to family circumstances, teachers were compelled to interpret the children’s unspoken inner states and situations. This difficulty in judgment contrasts with healthcare and welfare settings, where diagnostic and familial data are often shared through medical records. In schools, familial information is difficult to access without parental consent, leaving teachers to independently determine the necessity of support based on limited observational cues. Consequently, teachers often perceive isolation in their decision-making and experience hesitation or anxiety before initiating supportive actions.

Previous studies enrolling children with parents with cancer have repeatedly reported that family stress manifests psychological and behavioral changes in children [4, 9, 25]. These changes are most likely to be observed early in daily school interactions. Nonetheless, the lack of information often impedes a confident interpretation of these subtle changes as indicators of the need for continuous support, revealing the limitations of school-based judgment alone. Parrillo et al. investigated the long-term impact of parental cancer on children’s academic and emotional development and reported that inadequate collaboration between schools and healthcare institutions contributed to delays in intervention [23]. Furthermore, the teachers’ hesitation was shaped by concerns that support might be perceived as excessive interference in the children’s or their families’ private lives, reflecting underlying ethical dilemmas in balancing support with appropriate boundaries.

Therefore, healthcare professionals must understand the unique judgment structures and constraints of these educational settings. Additionally, they must work toward building collaborative systems in which teachers’ observational insights, uncertainties, and hesitations can be shared. Given that early recognition of children’s psychological responses can potentially improve the quality of support for the entire family [26], cooperative frameworks complementing the wavering judgment of teachers are crucial.

### Regulating emotional conflicts that hinder the provision of support: dilemmas at the boundary between education and family

Despite the changes detected in children, the teachers had various doubts and conflicts before taking supportive action. Subcategories such as hesitation to probe into family matters and the desire to respect the children’s own timing of disclosure reflected ethical hesitation regarding the appropriateness of the intervention. Teachers continuously questioned whether their involvement might become excessive, and they were torn between respecting the privacy of the family and upholding an educational stance that values children’s readiness to speak.

Furthermore, as indicated in subthemes such as anxiety stemming from a lack of certainty in judgment and barriers to consulting with colleagues or the school system, even when teachers intuitively perceived a need for support, they were burdened by insufficient information to justify their judgment and the ambiguity of consultation pathways. These structural factors suggest limitations in educational settings, where judgment and actions tend to rely heavily on individual discretion. Cautiousness and conflicts surrounding support thus represent teachers’ wavering role recognition, which is shaped by the interplay of multiple contextual and ethical factors.

In nursing practice related to family support in the context of cancer, nurses experience ethical dilemmas when initiating support [27], suggesting that similar challenges arise in educational settings. Moreover, children in families affected by parental cancer are vulnerable to psychosocial risks, and timely intervention is essential [28]. Therefore, teachers’ hesitation toward support should not be viewed negatively; rather, it can be interpreted as a manifestation of sensitive consideration and ethical responsibility toward the child and their family. Therefore, fostering a dialogical culture within schools, where teachers can consult and share their uncertainties, is a crucial first step in providing support. In addition, even when healthcare professionals do not directly intervene, their involvement as supportive partners with whom teachers can share doubts may help alleviate teachers’ anxieties and facilitate the initiation of support.

These emotional conflicts are particularly pronounced within the Japanese sociocultural context, where cultural norms emphasizing privacy, restraint in disclosing personal or family matters (“uchi-soto” dynamics), and deference to authority figures shape both teachers’ behaviors and families’ willingness to share illness-related information [29]. In Japan, cancer has historically been associated with social stigma, and some families remain reluctant to disclose a parental diagnosis to schools out of fear of discrimination or pity, which further limits the information available to teachers and deepens their hesitation to make inquiries. The hierarchical nature of teacher–parent relationships in Japanese educational culture may also deter parents from proactively sharing health concerns, while simultaneously making teachers cautious about appearing to overstep boundaries of their professional role.

### Recognizing and reaffirming the role of teachers as educators in the provision of support: teachers’ multilayered supportive agency

Teachers recognized their roles in multilayered ways, as recipients of children’s disclosures, observers of subtle daily changes, and mediators who connect children to further support. Their stance of accepting children’s feelings within trusting relationships represented not mere problem identification but an educational ethic of respecting the child’s timing of disclosure and engaging with support accordingly. Moreover, teachers were aware of the limitations inherent in their position as school educators, such as restricted access to medical and welfare expertise and the difficulty of directly intervening in the family matters. Nevertheless, they expressed expectations and attempted to collaborate with external professionals at appropriate moments. Teachers found value in continuously monitoring signs of stress or anxiety expressed by children and sustaining their attentiveness to such changes over time.

These practices resonate with the framework of family centered care in nursing [30, 31], emphasizing comprehensive support that considers children’s psychological and social contexts in the school setting. As supportive approaches integrating educational and nursing perspectives, the involvement of school nurses and the development of collaborative structures with healthcare institutions are particularly notable, suggesting new domains of practice at the interface between education and healthcare.

For nursing education, it is essential to foster an understanding of how children, especially those at heightened psychosocial risk, such as those with a parent with cancer, live their daily lives within their communities and build relationships with different supporters. Cultivating the capacity to support such children requires curricula that incorporate interprofessional collaboration with educators. Moving forward, it is crucial to establish collaborative support models grounded in everyday life, in which teachers and nursing professionals mutually understand and respect each other’s roles and boundaries.

Concrete mechanisms to operationalize such collaboration may include: (1) regular case conferences involving homeroom teachers, school nurses (yogo kyoyu), school counselors, and community healthcare professionals to review children identified as potentially at risk; (2) established referral pathways whereby teachers can formally refer concerns to school nurses or external social workers without requiring definitive diagnostic certainty; and (3) liaison roles for oncology nurses or hospital social workers to provide consultative support to schools when a parent is undergoing cancer treatment. With respect to managing confidentiality, schools and healthcare institutions should develop clear protocols specifying what information can be shared, under what conditions, and with whom, including mechanisms for obtaining parental consent for inter-institutional information sharing. Regarding teacher training, professional development programs should include the following components: recognition of psychosocial risk indicators in children experiencing family illness; culturally sensitive communication strategies that account for Japanese norms around privacy and illness disclosure; awareness of cancer-related stigma and its effects on family communication; knowledge of internal and external referral resources; and basic principles of collaborative care between educational and healthcare settings. Implementing such training as part of ongoing professional development, rather than as a one-time intervention, would enable teachers to respond more confidently and effectively to the complex needs of children affected by parental cancer.

### Limitations

This study had three main limitations. First, this qualitative study focused on the process through which teachers determined the necessity of support and did not explore the actual interventions implemented as a result of teachers’ judgment or assess their outcomes for children and families. Thus, the findings reflect the subjective perceptions and narratives of teachers, which may not necessarily correspond to actual support practices or their impact.

Second, although all participating children shared the background of having parents with cancer, details on parental disease stage, treatment trajectory, and prognosis were not identified. As such, variations in teachers’ judgments related to these clinical differences could not be fully analyzed. Future studies should adopt multilayered designs that compare children’s psychosocial experiences and support needs based on cancer type, disease progression, and treatment stage. Furthermore, clarifying the differences in perspectives on support between healthcare and education and providing practical examples of collaboration represent important future directions. Although healthcare and education share the goal of supporting children’s development and well-being, they diverge in their starting points, rationales, and objectives: healthcare typically centers on diagnosis and treatment, whereas education emphasizes daily observation and relational support. Understanding these distinctions while identifying common goals is essential for building effective interprofessional collaboration, thereby enabling more appropriate support decisions for children and families facing the everyday difficulties associated with parental cancer.

Third, all the participants were elementary school teachers in Akita City, a specific region of Japan. The unique educational culture and availability of local healthcare and welfare resources may have influenced teachers’ judgment and role recognition. Thus, the generalizability of these findings to other regions is limited. Comparative studies should be conducted across multiple regions and municipalities with differing educational and healthcare resources to distinguish the commonalities and context-specific characteristics in teachers’ judgment. Such studies may contribute to the development of flexible, locally responsive, collaborative support systems. Additionally, this study did not systematically examine the presence or content of institutional policies guiding teachers and parents in situations involving parental illness. In Japan, while national guidelines for school health management exist (e.g., those issued by the Ministry of Education, Culture, Sports, Science and Technology), specific protocols addressing how teachers should respond when a child’s parent has cancer are largely absent at the school level. Future research should examine how institutional policies shape teachers’ decision-making and what policy frameworks are needed to support consistent, appropriate responses across different school settings.

## Conclusions

Elementary school teachers recognized subtle signs of distress and changes in children with parents with cancer and, while experiencing uncertainty and ethical dilemmas, gradually acknowledged their professional role in providing support. The complexity of support-related judgments in educational settings underscores the need for close collaboration with healthcare and nursing professionals. Teachers’ daily observations of children provide a valuable basis for timely support from healthcare providers. Moving forward, strengthening systems that facilitate collaboration between education and healthcare, alongside educational initiatives that foster a mutual understanding of professional roles, will be essential for establishing comprehensive support for children with parents with cancer.

## Supplementary Information

Supplementary Material 1.

## Data Availability

The data and materials used for analysis and to support the conclusions of this study are available from the corresponding author upon reasonable request.
