# Supplementary material for: Assessment process followed by elementary school teachers in Japan for assessing the support needs of children of parents with cancer: a semi-structured interview
Source: BMC Pediatr. 2026 Mar 21;26:582. doi: 10.1186/s12887-026-06747-0 (PMC13282854; doi:10.1186/s12887-026-06747-0)
Supplement: Supplementary file 1 — Supplementary Material 1. [file 12887_2026_6747_MOESM1_ESM.docx]

**Semi-Structured Interview Guide**

**Demographic Information**

Prior to the interview, participants were asked to provide the following demographic information:

1. Age
2. Years of teaching experience
3. Position (e.g., classroom teacher, administrator, school nurse)
4. Personal or family experience with cancer
5. Experience in cancer education
6. Number of cases in which they had provided support to children with parents with cancer

**Semi-Structured Interview Questions**

The semi-structured interview included the following open-ended questions:

1. In what situation did you first feel that ‘support might be needed’ for children with parents with cancer, and what aspects of the children’s condition or changes were most striking at that time?
2. What were the decisive reasons or contextual factors for considering the necessity of support? Please describe any emotions or hesitations you felt at that time.
3. How did you, as a teacher, interpret any discomfort or signs you noticed while interacting with the children?
